# Draft Genome Sequence of *Lactobacillus plantarum* Strain A6, a Strong Acid Producer Isolated from a Vietnamese Fermented Sausage (Nem Chua)

**DOI:** 10.1128/genomeA.00987-17

**Published:** 2017-10-12

**Authors:** Aida Golneshin, Mian-Chee Gor, Thi Thu Hao Van, Bee May, Rob J. Moore, Andrew T. Smith

**Affiliations:** a School of Science, RMIT University, Melbourne, Victoria, Australia; b Griffith Institute for Drug Discovery, Griffith University, Nathan, Queensland, Australia; c Edlyn Foods Pty. Ltd., Melbourne, Victoria, Australia; d Griffith Sciences, Griffith University, Brisbane, Queensland, Australia

## Abstract

*Lactobacillus plantarum* strain A6, a strong acid producer, was isolated from a Vietnamese fermented sausage (nem chua). Here, we report the genome sequence of this strain (3,368,579 bp).

## GENOME ANNOUNCEMENT

Lactic acid bacteria (LAB) are microorganisms widely used in food fermentation as starter cultures because of their beneficial influence on food preservation and the organoleptic characteristics that they create in fermented foods (1, 2). Food preservation is associated with rapid acidification through the production of lactic acid, which in turn inhibits the growth of food spoilage bacteria (3, 4). Other metabolites, including antimicrobial peptides, vitamins, enzymes, and exopolysaccharides, are important for production of the final food product and for probiotic effects that can be beneficial for human health (5, 6). The use of *L. plantarum* in food fermentation has received recent attention, and attempts to systematically define genetic loci associated with wider probiotic properties have been made (7). Genome data for potential new probiotic stains (8) will assist in this task, in comparison to some well-established model genomes (e.g. that of *L. plantarum* WCFS1) (9). *L. plantarum* strain A6 isolated from the Vietnamese fermented sausage nem chua has been shown to be a strong acid producer, having potential for applications in food fermentation (10). Here, we report the draft genome sequence of *L. plantarum* strain A6.

The A6 genome was sequenced using the Illumina MiSeq sequencing platform (RMIT University, Australia) with 2 × 300-bp paired-end reads. The genomic library preparation was performed using the Nextera XT DNA library preparation kit. The *de novo* assembly was performed on the A5-miseq pipeline, an open source for genome assembly from Illumina data (11), and consisted of 66 contigs with 150-fold coverage. These contigs were analyzed using the NCBI Prokaryotic Genome Annotation Pipeline (PGAP) for gene prediction, and the gene sequences obtained were compared using Rapid Annotation of microbial genomes using Subsystems Technology (RAST) (12, 13).

PGAP revealed that the genome size of *L. plantarum* A6 is 3,368,579 bp in length, with a GC content of 44.2%. PGAP identified 3,218 genes, including 3,028 coding sequences, 92 RNAs, and 98 pseudogenes. There are 22 rRNAs, 66 tRNAs, and 4 noncoding RNAs (ncRNAs). RAST identified 3,261 coding sequences and 91 RNAs, including 68 tRNAs and 23 rRNAs. Several native plasmids are likely to be present in the strain; however, further investigation is required to define these precisely.

A comparison of the A6 genome with that of the previously published *L. plantarum* strain B21 (GenBank accession number NZ_CP010528) (8) isolated from the same source revealed that 2,954 out of 3,261 genes (90.6%) of A6 aligned well with those of B21 with a mean identity of 95.6%. A total of 307 genes were unique to A6. In addition, 2,896 out of 3,261 genes of A6 showed a mean identity of 95.8% to the corresponding genes from *L. plantarum* WCFS1 (GenBank accession number AL935263), a reference strain isolated from human saliva (9). There were 365 unique genes in the A6 genome that did not align to WCFS1.

The significant number of transpositions relative to its nearest neighbors, B21 and WCFS1, confirm the well-known plasticity of the LAB genome and this Vietnamese isolate.

### Accession number(s).

The complete genome sequence of *L. plantarum* strain A6 has been deposited in NCBI GenBank under the accession number LRUO00000000.
